# Efficacy of Medical Ozone as an Adjuvant Treatment in Dogs with Intervertebral Disc Protusions—A Retrospective Study

**DOI:** 10.3390/ani13233717

**Published:** 2023-11-30

**Authors:** Miriam Portero, Luis Villalonga, Mercedes Hernández, Carmen Pérez Díaz

**Affiliations:** 1 Department of Small Animal Medicine and Surgery, Hospital Clinico Veterinario Complutense, Veterinary Faculty, Universidad Complutense de Madrid, Avda. Puerta de Hierro s/n, 28040 Madrid, Spain; cperezdiaz@vet.ucm.es; 2 Hospital Veterinario, Puchol. C/Sauceda, 8, 28050 Madrid, Spain; luisvillalongarodriguez@gmail.com; 3 Clínica Veterinaria DRAMP, C/Juan XXIII, 69, 28500 Madrid, Spain; bertaviles@gmail.com

**Keywords:** ozone, intervertebral disc protrusion, veterinary medicine, dog, pain, quality of life

## Abstract

**Simple Summary:**

In humans, intervertebral disc degeneration is a chronic condition, the course of which leads to pain and inability to walk. Similarly, in dogs, intervertebral disc protrusion is a chronic pathology with a major impact on quality of life (QL). Surgical treatment is uncommon and conventional medical treatment is sometimes contraindicated due to the patient’s comorbidities, or is ineffective. New adjuvant strategies are being sought to improve patients’ QL. Of these, treatment with medicinal ozone (MO) is gaining prominence in recent years due to its analgesic, anti-inflammatory, and antioxidant effects. A retrospective clinical study was carried out in patients diagnosed with intervertebral disc protrusions in which MO was used as an adjuvant treatment to conventional medical treatment (physical rehabilitation, analgesics, anti-inflammatory drugs) in an attempt to improve their QL. A total of 21 dogs were included in this study. All patients showed an improvement in neurological signs and spinal pain with a consequent improvement in QL. No serious adverse effects were observed. Ozone therapy could be a potential therapy to be consider as a complementary treatment in dogs with intervertebral disc protrusions where surgery is not possible and/or medical treatment is insufficient. Prospective studies are necessary.

**Abstract:**

Ozone-therapy is used in humans as a coadjutant treatment in intervertebral disc diseases due to its analgesic, anti-inflammatory and antioxidant effects. References in dogs are scarce and limited to clinical cases (intradiscal/paravertebral infiltrations). The aim of this study was to assess the use of medical ozone (MO) as an adjunctive treatment in dogs with intervertebral disc protrusions (Hansen Type II/Chronic). A retrospective study was conducted in dogs diagnosed with intervertebral disc protrusions by MRI/CT in which MO was used as an adjuvant therapy to conventional medical treatment. Neurological examination and quality of life (QL) at the beginning and end of study were recorded, as well as posology and possible side effects. A total of 21 patients of different breeds and sex with a mean age of 12 years were included in this study. Results showed pain relief (7 ± 3 days) and improvement of neurologic signs (11 ± 9 days) with a consequent increasement in QL (13 ± 9 days). Thirteen out of the twenty-one patients (62%) showed a complete remission of the clinical signs. No serious adverse effects were observed. Medical ozone could be a potential complementary therapy to medical treatment in dogs with intervertebral disc protrusions. Prospective studies are necessary.

## 1. Introduction

Intervertebral disc disease (IVDD) in veterinary medicine encompasses different entities that affect the intervertebral discs causing different degrees of spinal cord injury. Spinal cord injury originating in the course of IVDD is the consequence of direct damage to cell membranes and microvascularization (primary injury) that will subsequently trigger secondary injury (systemic, extra-, and intracellular alterations). Secondary injury includes vascular effects (progressive decrease in perfusion and necrosis of the injured section with loss of self-regulation), biochemical effects (increase in intracellular calcium, production of free radicals, increase in excitatory amino acids, and ischemia associated with endorphins) and the inflammatory response to damage in the central nervous system [1,2].

It has been demonstrated that there is a significantly different status of oxidative stress between normal and degenerated intervertebral disc tissues, which indicates that immunity abnormality plays an important role in the pathogenesis of disc disease [3]. This oxidative stress induces inflammation, leading to nucleus pulposus cell nutritional metabolism disorders, apoptosis, decline of cell number, senescence, and autophagy [4]. The intervertebral disc is invaded by granulation tissue with blood vessels that bring cells from the immune system that release inflammatory mediators, generating even more oxidative stress and reactive oxygen species (ROS), promoting a vicious cycle [5,6].

In humans, intervertebral disc degeneration is a progressive and invalidating disease involving high socioeconomic cost with a multifactorial etiology and is the consequence of failure in tissue repair. In its chronic form, it initiates with mild pain, which gradually intensifies, often leading to an inability to walk [7,8]. Similar pathological changes have been reported in degenerated intervertebral discs from humans and dogs. The clinical presentation, macroscopic and microscopic appearance, diagnostics, and treatment of intervertebral disc degeneration and herniation are similar in humans and dogs. Decompressive surgery and spinal fusion are possible treatments in both humans and dogs, with fusion less often used in the latter species. The most important clinical/anatomical difference between both species is the relationship between the length of the vertebral column and the spinal cord. Usually in dogs, the spinal cord terminates between the two last lumbar vertebrae (L6-L7), while in humans, it terminates between the first two lumbar vertebrae (L1-L2). Therefore, the usual disc herniation sites in the terminal lumbar area (L3-L4, L4-L5, L5-S1) in humans result in only nerve roots of the cauda equina being affected, while the usual thoracolumbar or lumbar localization of disc herniations in dogs will directly compress the spinal cord [9]. Therefore, pain in humans with chronic discopathies is mainly associated with nerve root compression, without forgetting the other multifactorial components such as facet or discogenic pain especially in low back pain [7,8]. However, in dogs, the main source of pain is stimulation of the nociceptors of the dorsal longitudinal ligament as a consequence of spinal cord compression [10], although they may also suffer from radicular pain [11].

The overall appearance of both healthy and degenerating intervertebral discs from humans and dogs has been found to be similar, except that the canine intervertebral disc represents about 20% of the length of the spine, while in humans, the discs represent 25% [12]. All five stages of degeneration described in human have also been described in dogs [13]. Gross pathology of intervertebral disc degeneration in dogs and humans showed many similarities, but the cartilaginous endplates were significantly thicker and the subchondral cortices significantly thinner in humans than in dogs. The relative glycosaminoglycan content and metalloproteinase 2 activity in canine disc degeneration were similar to those in humans: metalloproteinase 2 activity increased and glycosaminoglycan content decreased with increasing severity of degeneration [14].

Dogs and other quadrupeds have higher vertebral bone densities than humans, indicating that axial compression stress is greater in dogs than in humans. The other mechanical loads are transformed by the surrounding musculature into axial compression, and eventually facet joint loads [9].

Advances in diagnostic imaging as well as genetic and histopathological studies have led to a better understanding of the possible etiologies triggering IVDD in veterinary medicine, as well as to a better classification [11]. Of these, intervertebral disc protrusions (Hansen type II/chronic) as a consequence of degeneration of the intervertebral disc are taking on great relevance in middle-aged and geriatric dogs due to the great impact on their quality of life (QL), as in humans. Clinical signs tend to reflect the chronic and slowly progressive nature of intervertebral disc degeneration associated with a chronic inflammatory state. The most common presentations are usually mild-to-moderate neurological deficits, not very painful myelopathy in older dogs, although pain may be present depending on the presence of nerve root compression [11], and the stimulation of the nociceptors of the dorsal longitudinal ligament [10].

In dogs with intervertebral disc protrusions, treatment is usually conservative since, being a progressive disease, surgical treatment should be performed before the onset of ataxia and paresis to avoid degenerative axonal lesions and demyelination, a rare occurrence since the diagnosis is usually made after months of evolution [10,15]. Medical treatment consists of combinations of relative rest, physical rehabilitation, and administration of analgesics, muscle relaxants, and anti-inflammatory drugs [16,17]. In the conservative treatment of cervical disc protrusions, the success rate is 48.9%, with a recurrence rate of 33% and 18.1% of failed treatments [17], similar results to thoracolumbar protrusions with 54.7% of successful cases, a 30.9% recurrence rate, and 14.4% of failed cases [16]. These figures reflect the complexity of medical treatment as the success or failure of treatment depends on many factors.

Ozone modulates oxidative stress through the activation of the Nuclear Factor Erythroid 2-Related Factor 2 (Nrf2) pathway in cells [18,19,20] and then induces the synthesis of cytoprotective proteins, which favors cell survival and improves the oxidative stress in the intervertebral disc degeneration [5,20]. Nrf2 appears to play an important role in the intracellular signaling pathways of inflammation. Indeed, the activation of the Nrf2-antioxidant signal could attenuate the nuclear factor kappa-light-chain-enhancer of activated B cells (NF-κB), a key regulator of the inflammatory response and muscle atrophy [21]. Ozone can reduce inflammation damage by regulating the inflammatory mediators (inhibition of prostaglandin E2 and phospholipase A2, IL 1, 2, 6, 8, 12, 15, and IFN-α) and the expression of cytokines (TNF-a) [22,23]. It also produces the oxidation of pain-mediating metabolites (release of immunosuppressive cytokines that induce anti-inflammatory and analgesic effects) and increasing local microcirculation and reduced venous stasis [21,24]. It is widely accepted that pain is a common symptom related to the inflammatory process and MO therapy could play a key role not only in the management of inflammation, but also in nociceptive perception and modulation. As for the analgesic use of MO, after the administration of MO, an increase in antioxidant molecules (serotonin and endogenous opioids) has been demonstrated, which would induce pain relief by stimulating the antinociceptive pathways [20,21].

Clinical studies in human medicine have demonstrated its efficacy in the oxidation of metabolites and pain mediators, muscle relaxation, enhancing local microcirculation with a reduction in venous stasis with the consequent reabsorption of spinal edema and improved mobility, reduction in disc volume by oxidation of proteoglycans, and reduction in root inflammation [5,25,26,27,28,29,30,31].

However, in veterinary medicine, references are very scarce and are limited to clinical cases of intradiscal administrations of medicinal ozone [32,33] and intramuscular paravertebral (PV) injections [34,35]. The aim of this study was to evaluate the use of MO as an adjunctive therapy to conventional medical treatment in dogs with intervertebral disc protrusions (Hansen Type II/Chronic) and its impact on patients’ QL. The hypothesis of this study was that MO as an adjuvant therapy could contribute to the improvement of dogs with intervertebral disc protrusions.

## 2. Materials and Methods

A retrospective clinical study was conducted between 2018 and 2022 by consulting the clinical records of dogs diagnosed in our hospital with intervertebral disc protrusions (Hansen Type II/Chronic) in accordance with the Canine Spinal Cord Injury Consortium (CANSORT-SCI) [3] that were treated with MO. Adjuvant MO therapy was introduced in the following: (1) dogs where surgical treatment was not indicated or refused by the owners and medical treatment was contraindicated or it was insufficient; (2) dogs where conventional medical treatment was contraindicated because of comorbidities; and (3) patients unresponsive to medical treatment. The study period was 12 weeks, considering the start of the study on the day of the first administration of MO (session 1) and on the last day of the study the session 16 (week 12). The final sample comprised a total of 21 dogs, 16 males and 5 females of different breeds, with a mean age of 12 years (range 7–14 years).

All subjects included in the study had informed consent to the therapy and its possible beneficial and adverse effects.

### 2.1. Inclusion Criteria

(a) Have a definitive diagnosis of at least one intervertebral disc protrusion (Hansen Type II/Chronic) by advanced imaging techniques [Magnetic Resonance Imaging (MRI) or Computed Tomography].
(b) Have neurological examination data at the beginning and end of the study.
(c) To have a record of the patient’s QL at the start and end of the study.
(d) Record of routes of administration, dosage, and possible side effects of therapy.
(e) Patients with previously prescribed medical treatment (anti-inflammatory drugs, analgesics, muscle relaxants, physical rehabilitation) at the start of MO treatment were included in the study as long as the medical treatment was not changed during the study period.

### 2.2. Exclusion Criteria

Dogs for which the addition MO to conventional medical treatment was not sufficient and analgesic rescue was needed were excluded from the study because the effects of analgesic rescue could mask the possible improvement of clinical signs by MO.

### 2.3. Data Collected from Medical Records

#### 2.3.1. Neurological Examinations

Pain was assessed by palpation in the affected vertebral region using a numerical scale (Table 1) and a value was assigned to the severity of clinical signs using a variant of the Modified Frankel Scale to also assess patients with cervical disc protrusions [17,36,37,38,39,40] (Table 2). Overall, 3 out of the 18 patients had a grade 4 on the Modified Frankel Scale, 8 grade 3, 5 grade 2, 1 grade 1, and 1 grade 0.

#### 2.3.2. Quality of Life Assessment

A QL survey referring to mobility and pain was developed to assess the evolution of patients (Table 3).

#### 2.3.3. MO Posology

- Routes of administration, concentration and volumes

Table 4 shows the routes of administration, concentration, and volumes used. The main local route of MO infiltration was PV (18/21; 86%) at increasing concentrations of 8–15 µg/NmL followed by subcutaneous (SC) (3/21; 14%), used at a concentration of 15 µg/Nml with a variable volume depending on the patient’s weight. In 2 patients, the SC route was used as the main route of administration due to the aggressiveness of the patients and the impossibility of performing PV infiltrations without sedation. In another patient, after developing an area of fibrosis in the regions of the paravertebral infiltrations, therapy was continued with subcutaneous infiltrations. In addition, rectal insufflation (RIO_3_) of MO at 3 mL/kg body weight at increasing concentrations of 20–35 µg/NmL was used as a systemic route in all patients (21 patients).

- Frequency of administration

Table 5 shows the frequency of administration of the ozone therapy sessions. The frequency of administration of medical ozone was as follows: first three weeks—three sessions per week, week 4 and 5—two sessions per week, week 6—one session, week 7—break, week 8—one session, and then one session per month or at the patient’s request. Each session must be separated from the previous one by at least 48 h.

The technique used for each route of administration is summarized in Appendix A.

### 2.4. Side Effects Recording

Events arising from the use of MO and the day of occurrence were recorded.

### 2.5. Statistical Analysis

Data analysis was performed using IBM SPSS Statistics 28.0.1.0. A descriptive statistical study was performed on qualitative variables (race, sex, co-morbidities, route of administration, and concentration of medicinal ozone used) and quantitative variables that included the mean, maximum, and minimum values (age, number of disc protrusions, time of illness from the onset of clinical signs to the start of treatment with MO, as well as pain, modified Frankel scale value, and QL at the beginning and end of the study and the times to achieve improvement in each variable). The times to achieve improvement in each variable were established according to the day on which the best score was obtained in the scales. In the analytical statistics, the Wilcoxon signed-rank test was used to analyze the evolution of the variables pain, modified Frankel scale value, and QL at the beginning and end of the study. The results were considered statistically significant if *p* < 0.05.

## 3. Results

### 3.1. Sample Size and Epidemiological Data

A total of 43 dogs were initially included in this study but 22 were excluded as they did not meet all the inclusion criteria. Overall, 3 out of the 22 excluded patients were not included in the final sample because it was necessary to increase the dose of opioids and/or gabapentin, despite being on treatment with MO. The remaining patients excluded from the study were due to incomplete medical histories that made it impossible to adequately evaluate the patients. The final sample therefore comprised a total of 21 dogs, 16 males and 5 females. Table 6 shows the summary of age, breeds, sex, comorbidities, and treatment at the time of study entry. Table 7 summarizes the location of the protruded disc, time of disease duration until the start of MO treatment, initial and final values of severity of neurological signs according to the Modified Frankel Scale, pain on examination and QL, as well as the time to reach maximum improvement in these three variables, and finally the patient’s recovery or not and adverse effects.

#### 3.1.1. Age and Clinical Signs

- The mean age of the patients was 12 years (range 7–14 years), the time of illness until the start of MO treatment ranged from 7 to 365 days with a mean of 54 days, and the number of discs protrusions per patient ranged from 1 to 9 with a mean of 3.
- Regarding the Modified Frankel Scale, 3 patients presented grade 4 (pain on palpation of the affected region), 8 grade 3 (ambulatory paraparesis/ataxia), 7 grade 2 (non-ambulatory paraparesis/tetraparesis), 2 grade 1 (paraplegia/tetraplegia with deep pain), and only 1 patient presented grade 0 (paraplegia without deep pain).
- Spinal pain was mild in 8 patients, moderate in 9 and severe in 4. The initial QL was good in 3 patients, regular in 11, and poor in 7.
- Statistically significant differences were found between the beginning and the end of the study for the variables Modified Frankel Scale (*p* = 0.00004), pain (*p* < 0.0001), and QL (*p* = 0.0001).

#### 3.1.2. Outcome

- In total, 24 patients started MO therapy; 3 out of these 24 dogs were excluded from the study before completing the full MO protocol because they needed rescue analgesia (opioid and/or gabapentin). All dogs included in the final sample (21 dogs) showed a statistically significant improvement at the end of the study in clinical signs according to the Modified Frankel Scale and in pain with a consequent improvement in overall QL. The median time for improvement in severity of clinical signs was 11 days (range 7–39 days), pain 7 days (range 7–16 days), and QL 13 days (range 7–41 days). Thirteen out of the twenty-one patients (62%) experienced complete recovery from the process (Grade 5 Modified Frankel Scale).
- In summary, 2 out of the 21 patients included in the study were euthanized due to progression of the neurological clinical signs leading to non-ambulatory paraparesis, and 11 patients died of diseases unrelated to the neurological condition under study. Currently, 8 patients remain alive from the study.

#### 3.1.3. Side Effects Recording

Side effects were observed in two patients. In both patients, an area of fibrosis was detected by palpation in the region of the PV infiltrations and confirmed by soft tissue ultrasound. There was a well-demarcated linear hyperechoic region within the epaxial musculature; two different patterns were identified—in one case there was a single focal lesion, whereas in a second one multifocal lesions of similar characteristics were present. In the latter patient, in addition to the area of fibrosis, a 5 cm subcutaneous hematoma was observed.

## 4. Discussion

In this study, the mean age of the patients was 10 years and 14 out of the 21 patients had comorbidities (chronic kidney disease, heart failure, non-steroidal anti-inflammatory drugs intolerance, and inflammatory bowel disease). In all the patients included in this study, a protocol of local PV and/or SC infiltrations was combined with a systemic route (RIO_3_) in order to enhance the local analgesic properties of MO [41] and achieve a systemic effect that could have an impact on the other pathologies. Systemic RIO_3_ was decided because of the ease of patient management [42,43].

All patients treated showed a statistically significant improvement at the end of the study in clinical signs according to the Modified Frankel Scale and in pain with a consequent improvement in overall QL possibly due to the antioxidant and anti-inflammatory effects of the MO. It has been demonstrated that there is a significantly different status of oxidative stress between normal and generated intervertebral disc tissues, which indicates that immunity abnormality plays an important role in the pathogenesis of disc disease [3]. Oxidative stress is due to the accumulation of ROS as a result of mitochondrial dysfunction; at the same time, the function of antioxidant enzymes in the intervertebral disc is inhibited, and the ROS clearance rate decreases. This oxidative stress induces inflammation, leading to nucleus pulposus cell nutritional metabolism disorders. This excess of ROS induces apoptosis and a decrease in cell number, senescence, and autophagy [4]. In this way, the intervertebral disc is invaded by granulation tissue with blood vessels and pathological innervation. The blood vessels bring cells from the immune system that release inflammatory mediators, generating even more ROS and promoting a vicious cycle [5,6]. Nrf2 is able to modulate inflammation through multiple mechanisms, such as the regulation of redox homeostasis and the suppression of pro-inflammatory genes, either directly or through the interaction with NF-κB. Inflammation increases local and systemic ROS level while ROS enhances inflammation. The Nrf2-mediated ROS-homeostatic control is able to break this vicious cycle. Nrf2 reduces inflammation by preventing the recruitment of RNA polymerase II to start gene transcription of pro-inflammatory cytokines IL-6 and IL1β [18,20,44]. Nrf2 activation exerts positive effects, especially on diseases that have oxidative stress and inflammation as primary etiopathological events as in dogs with disc protrusions that are the subject of our study [45,46].

The activation of Nrf2 is considered as a key factor for the efficacy of MO treatments. Ozone modulates oxidative stress through the activation of Nrf2 pathway in cells [18,19,20] and then induces the synthesis of cytoprotective proteins, which favors cell survival and improves the oxidative stress in the intervertebral disc degeneration [5,20]. MO therapy proved to be beneficial also for the treatment of spinal pain by activating various antioxidant pathways involving hypoxia inducible factor-1α nuclear factor of activated T-cells and activated protein-1 pathways [47]. All patients included in our study received systemic MO (RIO_3_). Systemic MO treatment in human healthy volunteers increased the levels of Nrf2 in peripheral blood mononuclear cells with consequent enhanced activity of superoxide dismutase and catalase [18]. Rectal insufflation of MO in human patients affected by multiple sclerosis increased Nrf2 phosphorylation and casein kinase 2 expression in mononuclear cells, thus improving the activity of antioxidant enzymes and reducing the levels of pro-inflammatory cytokines [48]. Therefore, it may be hypothesized that the therapeutic potential of Nrf2 activation as a consequence of mild ozonation relies on the capability of Nrf2 to maintain redox homeostasis; this would prevent DNA damage, preserve proteostasis, and improve mitochondrial function while suppressing acute and chronic inflammation [20].

The main local route of MO administration used in our study was PV infiltrations. PV infiltrations relieves pain in most patients by oxidizing pain-mediating metabolites (release of immunosuppressive cytokines that induce anti-inflammatory and analgesic effects) [5,28,49] and improves circulation and oedema resorption with consequent improvement in mobility [28,50]. The MO injected into the paravertebral muscle is rapidly dissolved in the interstitial water and quickly reacts with antioxidants; it has the same effect on the area near the disc. It can affect an area up to 3 cm from the injection site depending on the dose [31]. PV ozone injection has a better effect on the combination of biochemical effects in the muscle (improved oxygenation, correction of local acidosis, and loss of venous and lymphatic stasis). Muscle contraction/muscle spasm associated with pain is frequently observed in dogs with intervertebral disc protrusions. In this aspect, muscle relaxation is immediate after PV administration, decreasing the associated muscle contraction [31,50]. The literature states that injecting ozone gas into paravertebral muscles and discs can neutralize proteoglycan and the negative burden of sulphate side chains. This treatment reduces water retention and can decrease hernia volume [30,47].

Studies in human medicine have demonstrated, as in our study, the effectiveness of PV administration of MO in pain relief, with a higher success rate than treatment with only anti-inflammatory drugs (80% vs. 50%, respectively) [28]. In humans, a marked improvement has been observed during the first month of treatment [26,31]. Our study has also shown this tendency, with pain relief in the first two weeks of MO treatment, and an improvement in the QL during the first month of treatment.

The other local route used in the study was the SC route. There is no literature on the use of MO SC infiltrations in intervertebral disc protrusions in veterinary medicine. Its effectiveness has been demonstrated in a study with mice in which neuropathic pain was induced by a lesion in the sciatic nerve and a normalization of proinflammatory caspases expression and a decrease in pain signs were detected after a single subcutaneous administration of medicinal ozone [51]. In veterinary medicine, a limiting factor when administering any treatment that requires manipulation of the patient is patient’s behavior. Although PV infiltrations performed with proper technique are usually well tolerated in dogs, the pain threshold and especially the stress of the patients can make them painful. SC infiltrations are a great alternative to PV infiltrations because of their rapidity and less painful sensation [52]. In this study, SC infiltrations were used in two aggressive patients from the start of treatment and in one patient after he developed fibrosis the area of the PV infiltrations. In all of them, the effect was satisfactory, with a reduction in pain similar to PV infiltrations. It would be interesting to carry out a study using only local SC infiltrations to properly assess their efficacy.

The median time for improvement in severity of clinical signs was 11 days (range 7–39 days), for pain 7 days (range 7–16 days), and for QL 13 days (range 7–41 days). Two patients took longer to respond to treatment and their improvement in QL was not as marked as in the other patients. One of these patients was diagnosed with hemangiosarcoma of the spleen one month after the end of the study. This type of tumor is very aggressive and consumptive and often has associated inflammation [53]. It is likely that this tumor-associated inflammation benefited from the systemic effect of rectal insufflations to the detriment of their action on the spinal cord [54]. The other patient’s comorbidities included chronic kidney disease that flared up after an acute gastroenteric process. As in the previous case, it is likely that the systemic effect of MO was reduced at the spinal cord level due to the high oxidative stress resulting from the exacerbation of his chronic kidney disease.

The 9 patients with rapid resolution of clinical signs (7 days) are among the patients with the shortest time of illness (mean 30 days; range 7–90 days). The earlier a correct diagnosis is made and appropriate treatment is instituted, the higher the success rate. In intervertebral disc protrusions, the time factor is crucial to avoid axonal degenerative lesions and demyelination [10,15]. Overall, 8 out of these 9 patients had a complete recovery of the process (resolution of neurological clinical signs; Modified Frankel Scale grade 5). The patient who did not recover was the only one who did not comply with the established MO treatment protocol. The action of MO is based on the phenomenon of hormesis and the pre-conditioning effect in which the response does not occur in a single dose value but in a range of doses achieved by repeated application to maintain and increase tolerance to the oxidative stress it induces in the organism [55,56]. Although this patient showed a clear improvement in the very short term, a complete response was not obtained, possibly due to non-compliance with the protocol and thus not achieving the pre-conditioning effect that could have led to the resolution of the process. This fact underlines the importance of performing the appropriate and complete therapeutic protocol to achieve the best possible effects.

There was a dog (patient 3) with a modified Frankel scale value of 0 (no deep pain) that recovered deep pain improving neurological signs, presenting at the end of study an ambulatory paraparesis (modified Frankel scale value of 3). There is insufficient scientific evidence that medical ozone alone can restore the functionality of demyelinated fibers. Functional connections delivering supraspinal input are present in some dogs with severe lesions and likely contribute to regaining independent ambulation after injury. Many dogs that persistently lack deep pain do still have functional connections across their lesions [57,58]. The implication of MO in the recovery of the deep pain in this patient cannot be demonstrated. More studies are needed in patients with absent deep pain.

Seven patients had 1 or 2 protruded discs, and in all of them, recovery was complete as opposed to patients with more than 2 herniated discs (14 patients), in which only six recovered full function. Each disc protrusion involves a direct aggression on the spinal cord with the consequent vascular, biochemical, and inflammatory effects associated with the secondary lesion, which in the case of intervertebral disc protrusions will not be as severe initially as in extrusions but will be perpetuated over time. This will result in, among other things, increased production of free radicals, progressive decrease in perfusion, and spinal cord ischemia. The greater the number of disc protrusions, the greater the spinal cord injury with more negative effects on the spinal cord and therefore less chance of full recovery. The same explanation could be applied to the severity of clinical signs and time of illness. Patients with a higher value on the modified Frankel Scale are those with a longer time of illness [2,15,16,59]. All this reflects once again the importance of early diagnosis of intervertebral disc protrusions in order to achieve the best therapeutic effects in general, and in this particular case, with the use of OM as an adjuvant treatment.

Sixty-two percent of patients experienced complete recovery (resolution of neurological clinical signs; Modified Frankel Scale grade 5), a much higher percentage than that observed in patients with conventional medical treatment alone (54.7% thoracolumbar protrusions/48.9% cervical disc protrusions) [16,17]. Laser therapy, hyperbaric oxygen therapy, electro-acupuncture, and electrical stimulation modalities are other adjuvant therapies to conventional medical or surgical treatment in patients with IVDD. The mechanisms of action of MO are similar to the effects of laser therapy (vasodilation, increased circulation, local oxygenation, and cell metabolism) [60], hyperbaric oxygen therapy (increase the tissue partial pressure of oxygen) [1] and electroacupuncture (it might have analgesic and anti-inflammatory effects as well as facilitating axonal repair and regrowth) [1,61].

However, the mechanisms of action of electrical stimulation modalities are different from those of medical ozone. One of the possible mechanisms of action suggested for electrical stimulation modalities is the recruitment of motor units in a nonselective, spatially fixed, and temporally synchronous fashion, which leads to heightened muscle fatigue. Another possible theory is the ionic alterations at the cellular level which lead to increased intracellular Ca^2+^ concentrations [1,62].

It is difficult to compare the results obtained in our study with other studies using these adjuvant therapies because most of the patients are post-surgical patients who have undergone hemilaminectomy [63]. Of these, the study by Hayashi and Matera, 2006 is the one with a similar population to ours. In this study, overall success rate was 88.5%, significantly higher than for the control group (58.3%). Success was considered to have occurred when a dog with grade 0.1 or 2 dysfunction was able to walk without assistance or had return of deep pain perception and when a dog with grade 3 or 4 dysfunction had pain control, and improvement in conscious proprioception and ataxia, or both [61]. If we were to consider these same parameters, our overall success rate would 95%, which is higher than the Hayashi and Matera study.

Sumida et al., 2023 have recently published a prospective, randomized, blinded study to compare the effects of ozone therapy applied at acupuncture points and electro-acupuncture to treat dogs with clinical signs of thoracolumbar discopathy [64]. In the ozone group, MO was administered by infiltration into acupuncture points with a concentration of 20 μg/mL, and a volume of 3 mL at each point. Acupuncture points used were Bladder 20 or Pi-Shu and Bladder 23 or Shen-Shu) (located in the paravertebral region), Lumbar Bai Hui, Stomach 36 or Hou-san-li, and in the space between the Kidney 3 or Tai-chi and Bladder 60 or Kun-lun points. In this study no statistically significant differences were found between the electroacupuncture group and the ozone group for the pain assessment score and numerical functional scale. However, when comparing these two variables in the same group at different times, there was a significant difference in both the electroacupuncture and ozone group, demonstrating the improvement of pain and neurological function using both treatments. In this study, both groups showed a rapid response in these variables within the first two weeks, which is in line with our study in which the median time for improvement neurological disfunction was 11 days and pain 7 days [64]. Although the route of administration of MO is different from Sumida’s study and ours (acupuncture points vs. paravertebral infiltrations), the results obtained seem similar.

Taking into account this success rate in this retrospective study, prospective studies on the use of MO in dogs with intervertebral disc protrusions with an adequate sample size are needed, as in many patients surgical treatment is not possible or conventional treatment is contraindicated or ineffective.

No patient showed signs of pain at the time of PV, SC, or RIO_3_ infiltrations. Adverse effects were observed in only two patients. One of them developed a region of fibrosis (probably caused by repeated infiltrations and not by MO itself) and a 5 cm subcutaneous hematoma at the PV injection site at week 22 of treatment. It is quite possible that the subcutaneous hematoma was secondary to subclinical disseminated intravascular coagulopathy as a consequence of a hemangiosarcoma of the spleen diagnosed one month after the end of the study [53]. The other patient had only mild fibrosis at one of the PV infiltration sites, secondary to repeated infiltrations and a history of dermatitis.

The main limitation of this study is its retrospective nature, the small sample size, and the lack of follow-up of the untreated patient to assess the long-term effectiveness of the treatment. More prospective randomized and controlled trials are needed to increase the reliability of MO in dogs with intervertebral disc protrusions. It would be interesting to establish the possible differences in combined MO treatment, as well as to carry out control MRI scans at the end of treatment to assess changes at the disc, spinal cord, paravertebral muscle, and subcutaneous tissue level.

## 5. Conclusions

The combined use of paravertebral/subcutaneous infiltrations with rectal insufflations of medical ozone could be an effective adjuvant treatment to conventional medical treatment in dogs with intervertebral disc protrusions (Hansen Type II/Chronic) as it improves neurological deficits, associated pain, and thus quality of life.

The combination of intramuscular paravertebral injections at increasing concentrations of 8–15 µg/NmL (volume 2–5 mL) and/or subcutaneous injections at concentrations of 15 µg/NmL (volume 10–20 mL) coupled with rectal insufflations at increasing concentrations of 20–35 µg/NmL (volume 3 mL/kg) appears to be an effective ozone therapy protocol in the treatment of chronic disc protrusions in dogs. Furthermore, it is easy to administer and does not require sedation or anesthesia of the patient.

Minor adverse effects (paravertebral fibrosis, subcutaneous hematoma) were observed in only two patients (11%), probably caused by repeated infiltrations and comorbidities and not by the medical ozone itself. In this study, medical ozone appears to be a safe technique.

Medical ozone could be a potential therapy to be considered as a adjuvant treatment in dogs with intervertebral disc protrusions, especially if they do not show an adequate response to conventional medical treatment or it is contraindicated. Prospective randomized and controlled trials are needed to properly assess their efficacy and potential use.

## Figures and Tables

**Table 1:** Spinal pain evaluation.

Spinal Pain	Value
No pain	1
Mild pain	2
Moderate pain	3
Severe pain	4

**Table 2:** Modified Frankel Scale.

Description	Grade
Normal	5
Pain without neurological signs	4
Ambulatory paraparesis/tetraparesis	3
Non-ambulatory paraparesis/tetraparesis	2
Paraplegia/tetraplegia with deep pain perception	1
Paraplegia/tetraplegia without deep pain perception	0

**Table 3:** Quality of life assessment.

Quality of Life	Description	Value
Excellent	No pain/weakness, optimal physical condition, exercise normally, normal life.	1
Good	Mild signs of weakness and pain.	2
Regular	Marked weakness, obvious signs of pain.	3
Poor	The dog is unable to exercise or move	4

**Table 4:** Routes of administration, concentration and volumes.

Route of Administration		Dosage		
		Volume	Concentration	Dose
RIO_3_	Session 1	3 mL/kg	20 µg/NmL	60 µg/kg
	Session 2		25 µg/NmL	75 µg/kg
	Starting session 3		30 µg/NmL	90 µg/kg
PV	Session 1	5 mL/point	8 µg/NmL	40 µg/point
	Session 2		10 µg/NmL	50 µg/point
	Starting session 3		15 µg/NmL	75 µg/point
SC	Small dog/single hernia	5 mL	15 µg/NmL	75 µg/point
	Large breed/multiple hernias	10 mL	15 µg/NmL	150 µg/point

RIO_3_: rectal insufflation; PV: paravertebral intramuscular; SC: subcutaneous.

**Table 5:** Frequency of administration.

Week	Session Number	Route of Administration
1	1	RIO_3_ + PV or SC
	2	RIO_3_
	3	RIO_3_ + PV or SC
2	4	RIO_3_ + PV or SC
	5	RIO_3_
	6	RIO_3_ + PV or SC
3	7	RIO_3_ + PV or SC
	8	RIO_3_
	9	RIO_3_ + PV or SC
4	10	RIO_3_ + PV or SC
	11	RIO_3_ + PV or SC
5	12	RIO_3_ + PV or SC
	13	RIO_3_ + PV or SC
6	14	RIO_3_ + PV or SC
8	15	RIO_3_ + PV or SC
12	16	RIO_3_ + PV or SC

RIO_3_: rectal insufflation; PV: paravertebral intramuscular; SC: subcutaneous.

**Table 6:** Summary findings I.

N	Age (Year)	Breed	Sex	Comorbidities	Treatment
1	13	Golden R.	M	AINE intolerance	Tramadol
2	12	W. Swiss G.	M	CKD	Acetaminophen, tramadol
3	12	Golden R.	M	CKD	ACEI, tramadol
4	8	Maltese	F	None	Meloxicam
5	7	Pug	M	AINE intolerance	Gabapentin
6	14	Chihuahua	M	CKD	ACEI, tramadol
7	13	Labrador	F	AINE intolerance	Acetaminophen, gabapentin
8	9	Rottweiler	M	AINE intolerance	Gabapentin
9	10	Mongrel	M	MMVD-B2	Pimobendan, ACEI, gabapentin
10	13	WHWT	F	None	Firocoxib, gabapentin
11	10	French Bull	M	None	Firocoxib, gabapentin
12	14	Yorkshire	M	CKD	Tramadol, pregabaline
13	10	French Bull	M	IBD	Acetaminophen, gabapentin
14	14	Mongrel	M	CKD	ACEI, tramadol
15	14	Mongrel	M	Leishmaniasis, CKD	ACEI, tramadol
16	9	Poodle	M	None	Meloxicam, gabapentin
17	12	Schnauzer	F	MMVD-B2	Pimobendan, gabapentin
18	9	Cocker sp	M	None	Firocoxib, pregabalin
19	13	Weimaraner	F	None	Firocoxib, gabapentin
20	14	Greyhound	F	None	Firocoxib, gabapentin
21	13	Mongrel	M	CKD	Tramadol

N: patient; F: female; M: male; AINE: non-steroidal anti-inflammatory drug; CKD: chronic kidney disease; MMVD-B2: myxomatous mitral valve disease stage B2; ACEI: Angiotensin converting enzyme inhibitor; IBD: inflammatory bowel disease.

**Table 7:** Summary findings II.

N	Location of Protruded Disc	TD (d)	MFS_o_	MFS_f_	tMFS (d)	Pain_o_	Pain_f_	tPain (d)	QL_o_	QL_f_	tQL (d)	Rec	SE
1	T12-L4, LS	120	2	3	21	2	1	14	3	2	41	+	-
2	T2-L5, LS	120	2	3	39	3	1	16	4	3	34	-	+
3	T13-L4, LS	180	0	3	15	2	1	15	4	1	15	-	-
4	T12-L1	90	2	5	7	3	1	7	3	1	7	+	-
5	T12-L1, LS	365	2	3	21	3	2	7	3	1	7	-	-
6	C2-C7	90	2	3	28	4	2	7	4	3	34	-	-
7	T12-L2, L3-L6	40	4	5	7	2	1	7	2	1	14	+	-
8	LS	30	3	5	7	3	1	10	3	1	17	-	-
9	T12-L1	7	4	5	7	3	1	13	3	1	13	+	-
10	T13-L1	365	3	5	21	2	1	7	3	1	21	+	+
11	T13-L2, LS	300	3	5	21	2	1	7	3	1	21	+	-
12	C2-C5	365	2	5	28	2	1	7	4	1	28	+	-
13	T11-L2, LS	60	3	5	7	2	1	7	3	1	7	+	-
14	T13-L1, L3-L5	30	3	5	21	4	1	3	4	1	7	+	-
15	T13-L3, LS	90	3	5	14	3	2	7	2	1	21	-	-
16	L1-L3	365	4	5	21	4	1	3	3	1	7	+	-
17	T13-L1, LS	90	3	5	7	3	1	7	2	1	14	+	-
18	L2-L3	60	3	5	7	4	1	7	3	1	7	+	-
19	C2-C4, C5-C7	90	1	3	7	3	1	7	4	2	21	-	-
20	L1-L3, LS	90	1	3	7	2	1	7	4	1	14	+	-
21	T13-L2, L4-L5	90	2	3	7	3	1	14	3	2	21	-	-

N: patient; TD: time of disease; d: days; MFSo: Modified Frankel Scale at the beginning of the study; MFSf: Modified Frankel Scale at the end of the study; tMFS: time to improve the grade of the Modified Frankel; Paino: pain scale at the beginning of the study; Painf: pain scale at the end of the study; tPain: time to improve the pain scale; QLo: initial quality of life; QLf: final quality of life; tQL: time to improve quality of life; Rec: recovery; SE: side effects; C: cervical vertebrae; T: thoracic vertebrae; L: lumbar vertebrae; LS: lumbosacral vertebras.

## Data Availability

Data are contained within the article.

## Appendix A

The MO mixture was obtained using an Ozonobaric P-SEDECAL^®^ medical ozone generator.

Rectal insufflation technique: a 20, 50 (Terumo^®^, Terumo Europe N.V., Leuven, Belgium) or 100 mL latex-free 3-body syringe (Omnifix^®^, BBraun Medical AG, Escholzmatt, Switerland) was used depending on the size of the patient. Once the MO mixture is generated, the syringe is connected to an 18 cm vaginal probe (Klinik Health^®^, Izasa Hospital, L’Hospitalet de Ll, Barcelona). Administration is performed with the patient in lateral decubitus or stationary position, introducing the catheter through the anus up to the level of the rectal venous plexus.

Paravertebral intramuscular injection technique: 10 mL latex-free 3-body syringes (Terumo^®^, Terumo Europe N.V., Leuven, Belgium) and 25G needles (BD Microlance 3^®^, NIPRO MEDICAL EUROPE, Mechelen, Belgium) were used. The patient was placed in sternal decubitus or stationary position and the hair in the area to be infiltrated was shaved and disinfected. Subsequently, the intervertebral spaces with the herniated discs were located by palpation of the ribs and spinous and transverse processes and the MO was infiltrated perpendicular to the rachis 2–3 cm from the spinous processes in the paravertebral musculature close to the intervertebral spaces with the herniated disc and in the spaces immediately anterior and posterior.

Subcutaneous injection technique: 10 mL latex-free three-body syringes (Terumo^®^, Terumo Europe N.V., Leuven, Belgium) and 25G needles (BD Microlance 3^®^, NIPRO MEDICAL EUROPE, Mechelen, Belgium) were used. With the patient at the station, the intervertebral spaces to be treated were located by palpation and the MO was injected into the subcutaneous tissue dorsal to the spinous processes in 2 (in single hernias) or 4 points (in multiple hernias) close to the intervertebral spaces with the herniated disc.
